# Artificial intelligence in neurovascular surgery: advancing diagnosis, treatment, and outcomes

**DOI:** 10.3389/fsurg.2026.1841708

**Published:** 2026-06-18

**Authors:** Liping Li, Zhonghua Zhang, Lin Zong

**Affiliations:** Department of Anesthesiology, Jinling Hospital, Jinling School of Clinical Medicine, Nanjing Medical University, Jiangsu, China

**Keywords:** artificial intelligence, cerebrovascular disease, deep learning, machine learning, neurovascular surgery

## Abstract

Artificial intelligence (AI) is transforming neurovascular surgery by improving diagnostic accuracy, risk prediction, treatment planning, and patient outcomes. This narrative review examines AI across the continuum of cerebrovascular care, from initial diagnosis through intervention and long-term prognostication. We discuss how machine learning, deep learning, computer vision, and natural language processing are applied to diverse data sources including neuroimaging, electronic health records, and intraoperative inputs. AI algorithms augment clinical expertise in diagnosis by delivering high speed and precision for tasks such as detecting large vessel occlusions, characterizing aneurysm morphology, and differentiating hemorrhage subtypes. Beyond detection, AI models are increasingly used for risk stratification—predicting aneurysm rupture, functional recovery after stroke, and post-intervention complications. AI also shows promise in therapeutic decision-making through pre-operative simulation, robotic-assisted microsurgery, and intraoperative guidance systems, with preliminary evidence suggesting potential improvements in procedural safety and efficacy (though most intraoperative AI studies remain at the proof-of-concept or single-center retrospective stage). Despite these developments, challenges remain, including algorithmic bias, limited generalizability, lack of interpretability, data privacy concerns, and regulatory barriers. Successful deployment requires seamless workflow integration and a clear understanding that AI assists, not replaces, the neurosurgeon. The convergence of AI with precision medicine holds promise for personalized, data-driven care through synergistic human-AI collaboration.

## 1 Introduction

Cerebrovascular diseases—including intracranial aneurysms, acute ischemic stroke, and intracerebral hemorrhage—represent a leading cause of morbidity and mortality worldwide, imposing substantial clinical and economic burdens on healthcare systems (1). Timely and accurate diagnosis, precise risk stratification, and effective intervention are critical to improving patient outcomes. However, achieving these goals is often hindered by the complexity of neurovascular pathologies, the variability in clinical presentation, and the inherent limitations of human interpretation of imaging and clinical data. In this context, artificial intelligence (AI) has emerged as a promising tool in medicine, offering new capabilities to analyze multimodal data, uncover hidden patterns, and support clinical decision-making with speed and precision inaccessible through traditional methods (2).

Recent advances in machine learning, deep learning, and natural language processing have enabled AI systems to excel in tasks ranging from image-based detection of aneurysms on CT angiography (3–5) to prediction of hematoma expansion in intracerebral hemorrhage, and even real-time identification of cerebral vasospasm using transcranial Doppler signals (6). These technologies represent emerging concepts and are the subject of ongoing developments across the neurovascular care continuum—from early diagnosis and prognostication to preoperative planning, intraoperative guidance, and post-intervention monitoring (7–9). For instance, AI-driven models can predict aneurysm rupture risk based on morphological and hemodynamic features (10), while AI-guided robotic platforms are beginning to enhance procedural accuracy in endovascular therapy.

Despite this rapid progress, the clinical translation of AI in neurovascular surgery faces significant challenges, including algorithmic bias, lack of interpretability, data interoperability issues, and regulatory complexities (11). This review synthesizes current evidence on the role of AI across the neurovascular care pathway, underscoring its practical applications in diagnosis, risk prediction, and treatment. By critically evaluating both achievements and limitations, we seek to clarify how AI can serve as a powerful assistive tool—augmenting, not replacing, the clinician's expertise—to advance precision medicine and improve outcomes in neurovascular surgery. This is a narrative review that synthesizes the current literature on AI in neurovascular surgery. Given the broad and rapidly evolving scope, we did not aim to perform a full systematic review with meta-analysis.

## 2 Summarization in AI

**Figure 1:**
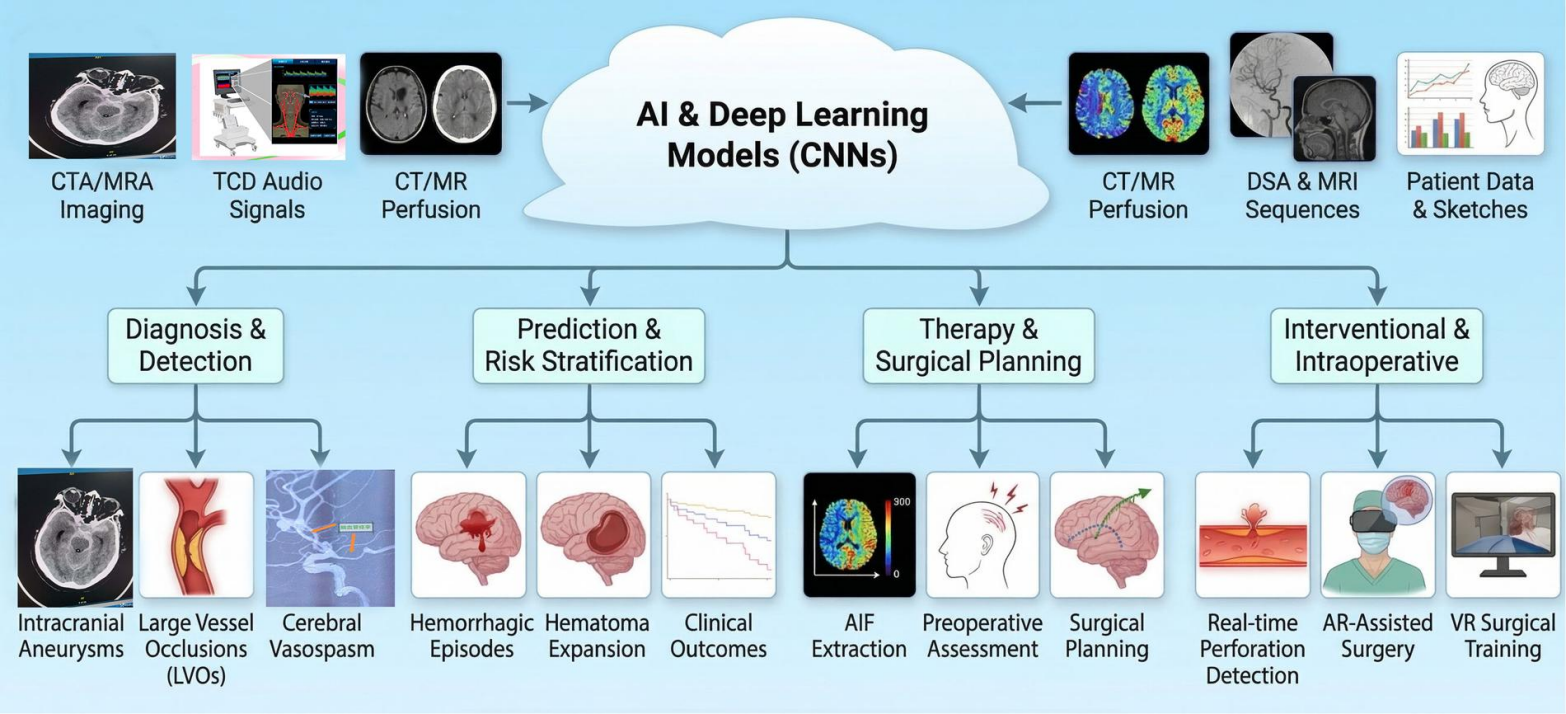
Overview of artificial intelligence applications in cerebrovascular disease management. This schematic illustrates the integration of AI and deep learning models, particularly CNNs, across the continuum of cerebrovascular care. The workflow is organized from left to right, beginning with multimodal imaging inputs—including CTA, MRA, and TCD audio signals—followed by four core application domains: (1) diagnosis and detection, (2) prediction and risk stratification, (3) therapy and surgical planning, and (4) interventional and intraoperative support. The yellow arrow points to the area of vascular spasm.

Recent advances in AI, particularly deep learning and convolutional neural networks (CNNs), have significantly transformed the diagnostic, predictive, and therapeutic paradigms in cerebrovascular disease management (Figure 1). For example, deep learning models have demonstrated high accuracy in detecting intracranial aneurysms and large vessel occlusions (LVOs) on CTA and MRA (see Section 4.1 for detailed performance metrics and critical appraisal). Comparative studies evaluating five distinct CNN architectures revealed that ensemble models incorporating attention mechanisms and multi-scale feature extraction demonstrated superior generalization on external validation datasets, with one ensemble CNN model reporting >95% sensitivity in a small subset of highly selected external validation studies (multi-center, *n* = 1,202) (12); however, such performance is not representative of the broader literature, where substantial heterogeneity and lower generalizability are common an external validation dataset. However, the same study noted that sensitivity varied across different CT scanner vendors (range: 89%–97%), highlighting the importance of device-specific validation before widespread implementation. Moreover, AI-enhanced perfusion imaging has streamlined the identification of arterial input functions (AIFs)—a critical step for accurate cerebral blood flow quantification—in both CT and MR perfusion studies. A deep CNN automatically extracted AIFs with high reproducibility, eliminating inter-observer variability and improving the reliability of penumbra-core mismatch assessment in ischemic stroke (13, 14). These capabilities directly support faster triage and treatment decisions, aligning with the “time-is-brain” principle in vascular neurology (11).

AI also plays a pivotal role in predicting clinical outcomes and guiding surgical planning. In moyamoya disease, deep learning models trained on digital subtraction angiography (DSA) and MRI sequences have been developed to not only recognize characteristic stenotic patterns but also predict the risk of future hemorrhagic episodes in adult patients, offering a proactive approach to intervention timing (15). Similarly, in intracerebral hemorrhage (ICH), AI models analyzing baseline non-contrast CT scans can predict early hematoma expansion—a key determinant of poor prognosis—with accuracy sufficient to guide decisions regarding surgical evacuation (16–19). This underscores AI's potential to integrate subjective patient-reported data into objective surgical planning.

Interventional and intraoperative applications of AI are equally transformative. During endovascular thrombectomy, a spatio-temporal deep learning model demonstrated the ability to detect and localize intracranial vessel perforations in real-time DSA sequences, achieving an AUC of 0.93 in a retrospective analysis of 450 DSA sequences (17, 20). Of note, this was a proof-of-concept study using archived data from a single center; real-time intraoperative performance and impact on clinical outcomes have not yet been evaluated. Therefore, the claim of enhanced procedural safety remains speculative pending prospective, live-patient trials. Augmented reality (AR) systems integrated with AI further assist surgeons by differentiating feeding from draining vessels in arteriovenous malformations (AVMs), improving resection accuracy and reducing operative complications (21–23). Additionally, AI-driven virtual reality (VR) simulators are being used to assess and standardize neurosurgical training. One study employed machine learning to analyze six AI-derived metrics during repeated VR subpial resections, revealing distinct learning curves among medical students, junior residents, senior residents, and attending neurosurgeons—highlighting AI's utility in competency-based education.

Despite these advances, challenges remain. Many AI models suffer from limited external validation, dataset bias, and the lack of interpretability, which hinder clinical adoption (11). Furthermore, once AI shows a strong correlation with expert performance in retrospective studies (24), prospective randomized trials are needed to confirm its impact on hard clinical endpoints such as mortality or functional independence. Nevertheless, the integration of AI into neuroendovascular procedures—through enhanced image analysis, real-time decision support, and future robotic integration—holds immense potential to standardize care and improve patient outcomes across diverse healthcare settings (7).

Collectively, these innovations illustrate a paradigm shift toward data-driven, personalized cerebrovascular care. As AI systems become more robust, transparent, and integrated into clinical workflows, they are poised to enhance diagnostic precision, optimize therapeutic strategies, and ultimately improve long-term neurological outcomes for patients worldwide (1, 11).

## 3 Rationale and scope of this review

### 3.1 Literature search strategy

To ensure a systematic and reproducible synthesis of the literature, we conducted a structured search of PubMed for peer-reviewed articles published between January 2020 and December 2025. The search strategy combined keywords related to AI (“artificial intelligence,” “machine learning,” “deep learning,” “convolutional neural network,” “natural language processing”), neurovascular disease (“cerebrovascular,” “intracranial aneurysm,” “acute ischemic stroke,” “intracerebral hemorrhage,”), and clinical applications (“diagnosis,” “detection,” “prediction,” “segmentation,” “outcome,” “intervention,” “robotics,” “extended reality”). Only original research articles, systematic reviews, and meta-analyses written in English were included. We excluded conference abstracts, editorials, case reports, and studies without explicit AI model validation. Selection bias acknowledgment: While we did not exclude studies based on the direction or magnitude of their results, the search yielded predominantly studies reporting positive or high-performance metrics (e.g., sensitivity >90%, AUC >0.85). Neutral or negative findings—such as AI models failing to outperform simple clinical scores or showing poor external generalizability—were scarce in the peer-reviewed literature.

We conducted a targeted literature search in PubMed for peer-reviewed articles published between January 2020 and December 2025, using a combination of keywords related to AI (“artificial intelligence,” “machine learning,” “deep learning,” “convolutional neural network,” “natural language processing”), neurovascular disease (“cerebrovascular,” “intracranial aneurysm,” “acute ischemic stroke,” “intracerebral hemorrhage”), and clinical applications (“diagnosis,” “detection,” “prediction,” “segmentation,” “outcome,” “intervention,” “robotics,” “extended reality”). Additional relevant studies were identified through manual review of reference lists and by consulting recent high-impact journals. Because this is a narrative review with a broad scope and rapidly evolving literature, we did not perform an exhaustive multi-database search, formal risk-of-bias assessment, or meta-analysis. We acknowledge that the literature is dominated by positive results; this inherent selection bias is discussed in Section 8.7.

### 3.2 The burden of cerebrovascular disease

Cerebrovascular disease remains one of the leading causes of morbidity, mortality, and long-term disability worldwide, imposing a substantial burden on healthcare systems and societies. Stroke alone accounts for approximately 11% of global deaths annually, with ischemic stroke representing nearly 85% of all cases, while hemorrhagic subtypes—including intracerebral and subarachnoid hemorrhage—contribute disproportionately to case fatality rates (25–27). Beyond acute events, chronic cerebrovascular conditions such as moyamoya disease, cerebral aneurysms, AVMs, and progressive intracranial atherosclerosis significantly impair quality of life and increase the risk of recurrent neurological injury. The clinical manifestations range from transient ischemic attacks (TIAs) and focal neurological deficits to catastrophic outcomes like coma or death, often dictated by the speed and accuracy of diagnosis and intervention. Delays in identifying LVOs or predicting aneurysm rupture can result in irreversible neuronal loss, underscoring the critical need for rapid, precise, and accessible diagnostic tools. Moreover, post-stroke complications such as cerebral vasospasm following subarachnoid hemorrhage or delayed hematoma expansion in intracerebral hemorrhage further complicate management and worsen prognosis. Traditional imaging modalities—including non-contrast CT, CTA, MRA, and DSA—while indispensable, are subject to inter-observer variability, limited availability in resource-constrained settings, and interpretation delays that hinder timely therapeutic decisions. These challenges highlight an urgent unmet need for technologies capable of augmenting human expertise, standardizing assessments, and enabling earlier, more individualized interventions across the spectrum of cerebrovascular pathology.

### 3.3 Rationale and scope of this review

Despite the proliferation of AI applications in cerebrovascular disease, a comprehensive synthesis of their clinical validation, methodological rigor, and real-world implementation remains lacking. Despte reporting high internal performance, many studies face critical limitations—including insufficient external validation, dataset heterogeneity and bias, and incomplete reporting of model architecture and training protocols—that impede reproducibility and delay clinical translation. This review aims to critically evaluate the current state of AI in cerebrovascular medicine by focusing on three core domains: (1) diagnostic accuracy in detecting structural abnormalities (e.g., aneurysms, AVMs, stenosis); (2) predictive capabilities for clinical outcomes (e.g., hemorrhage risk, functional recovery, hematoma expansion); and (3) intraoperative and procedural assistance (e.g., real-time complication detection, augmented reality guidance). We emphasize studies that provide quantitative performance metrics—such as area under the receiver operating characteristic curve (AUC), sensitivity, specificity, and positive predictive value—and compare AI performance against expert clinicians where available. Special attention is given to prospective or multicenter validations, as these offer stronger evidence of generalizability. To contextualize the clinical impact, we summarize key findings in the following Table 1, which outlines representative AI applications, their technical approaches, performance benchmarks, and implications for patient management.

**Table 1:** Key AI applications in cerebrovascular disease: performance metrics and clinical impact.

Clinical application	AI model	Sample size	Performance metrics	Validation type	Clinical relevance	Risk of bias	Reference
Intracranial aneurysm detection (CTA)	Deep CNN	16,496/648	Sensitivity 95.7%, outperformed radiologists in small aneurysms	4 centers	Reduces missed diagnoses, enables screening programs	Retrospective; lower sensitivity for <3 mm aneurysms	(28)
Intracranial aneurysm localization (MRA)	3D ResNet	960/240	Sensitivity 94.7% (91.3–96.8), Specificity 91.2% (88.5–93.2)	Single-center	Facilitates preoperative planning and surveillance	Single vendor (3 T)	(29)
Cerebral vasospasm detection (TCD)	LSTM (recurrent neural network)	train/val/test: 698/87/87	AUC 0.88, Sensitivity 81%, Specificity 76%	Internal temporal cross-validation	Accelerates diagnosis post-SAH, guides nimodipine use	Retrospective; real-time performance not evaluated	(30)
Large vessel occlusion detection	Ensemble CNN	2,500/1,200	Sensitivity 96.2% (external validation)	multi-center	Shortens door-to-groin puncture time in thrombectomy	Variability across vendors (91–98% sensitivity)	(31)
Arterial input function extraction	Deep CNN (CTP/MRI)	1800	ICC 0.91 (MRI), Correlation 0.97 ± 0.04 (CTP)	6 centers	Improves the accuracy of penumbra-core mismatch assessment	Requires standardized acquisition protocols	(32)
Hematoma expansion prediction (ICH)	Machine learning on NCCT	(train/val/test: 602/129/129)	Clinically actionable risk stratification (AUC ∼0.87)	Single-center	Guides decisions on surgical evacuation vs. conservative care	Retrospective; spot sign data missing	(33)
Moyamoya hemorrhage risk prediction	Deep learning (various architectures)	Meta-analysis (total 4,795 patients across studies)	Pooled AUC 0.85 (95% CI: 0.82–0.89), pooled specificity 85%	Meta-analysis of multi-center studies	Prevents catastrophic rebleeding through early intervention	Retrospective; no prospective validation	(34)
Vessel perforation detection (DSA)	Spatio-temporal DL model	345 train/105 test	AUC 0.96 (95% CI: 0.91–0.99), Sensitivity 92%, Specificity 91%	Internal cross-validation	Enhances safety during endovascular procedures	Proof of concept	(20)

This review deliberately excludes speculative or purely technical studies lacking clinical correlation, focusing instead on AI systems with demonstrated or plausible pathways to bedside integration. By synthesizing evidence from peer-reviewed literature and highlighting both achievements and limitations—such as the need for prospective randomized trials and standardized reporting frameworks—we aim to provide clinicians, researchers, and policymakers with a balanced perspective on how AI is reshaping the landscape of cerebrovascular disease management and where future efforts should be directed to maximize patient benefit (35–37).

Several excellent reviews have summarized AI applications in cerebrovascular disease, including Chen et al. (1) on imaging, Alqadi et al. (11) on stroke tools, Senders et al. (24) on neurosurgery, Shlobin et al. (38) on LVO, and Jiang et al. (39) on deep learning in stroke. However, the present review offers several distinctive contributions. First, we provide an updated evidence base (2020–2025, with selected 2026 additions) that captures the most recent prospective and multicenter validation studies, many of which were published after earlier reviews. Second, we adopt a critical appraisal lens that systematically distinguishes internal vs. external validation, highlights negative findings and failure modes (e.g., decreased sensitivity for distal occlusions, false-positive alerts, lack of hard endpoint improvement), and avoids promotional language. Third, we explicitly integrate emerging technologies—extended reality simulation, augmented reality navigation, and robotic platforms—into the diagnostic and interventional AI pipeline, which previous reviews have treated separately. Fourth, we dedicate substantial discussion to clinical translation barriers, including workflow integration, regulatory hurdles, and publication bias, offering a realistic roadmap for adoption.

## 4 Fundamentals of AI and data types in neurovascular research

### 4.1 Key concepts in machine learning

**Figure 2:**
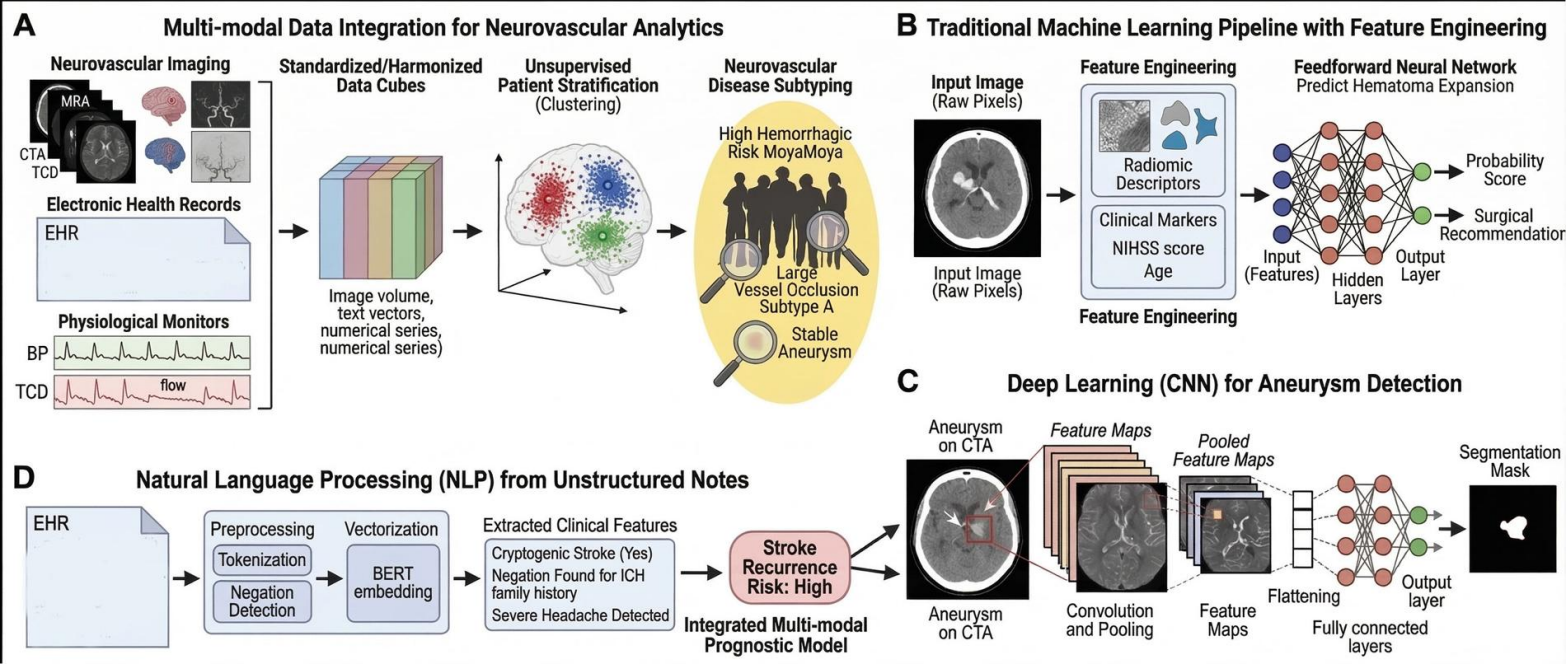
Fundamentals of artificial intelligence and data integration in neurovascular research. This schematic illustrates two complementary methodological paradigms for integrating multimodal data in neurovascular analytics. **(A)** Presenting the pathway from neurovascular imaging to predictive modeling, encompassing both traditional machine learning and deep learning approaches. Multimodal imaging data—including MRA, CTA, and TCD—are first transformed into standardized or harmonized data cubes, facilitating subsequent analyses. **(B)** Depicting a conventional ML workflow that relies on handcrafted feature engineering. **(C)** Presenting deep learning approach using CNNs that directly learns hierarchical feature representations from raw pixel data for tasks such as aneurysm detection. **(D)** illustrates the integration of unstructured clinical data via NLP.

Machine learning (ML), a core subset of artificial intelligence, has become instrumental in transforming neurovascular diagnostics and therapeutics by enabling computers to learn from data without explicit programming (Figure 2A). At its foundation, ML algorithms are broadly categorized into supervised, unsupervised, and reinforcement learning paradigms, each serving distinct purposes in cerebrovascular research. Supervised learning—where models are trained on labeled datasets such as annotated aneurysm images or outcome-labeled stroke cases—is particularly prevalent in clinical prediction tasks. For instance, logistic regression, support vector machines, and random forests have been successfully applied to predict early hematoma expansion in intracerebral hemorrhage using baseline non-contrast CT features, thereby informing decisions about surgical evacuation (40). Unsupervised learning, in contrast, identifies hidden patterns in unlabeled data and is increasingly used for patient stratification; clustering algorithms have revealed distinct subtypes of moyamoya disease based on hemodynamic profiles derived from perfusion imaging, which correlate with hemorrhagic risk (15). Reinforcement learning, though less mature in clinical deployment, holds promise for optimizing treatment sequences in complex endovascular interventions by simulating procedural decision trees.

The performance of ML models hinges critically on feature engineering—the process of selecting and transforming raw data into informative predictors (Figure 2B). In neurovascular applications, features may include radiomic descriptors (e.g., texture, shape, intensity heterogeneity from CT or MRI), physiological parameters (e.g., blood pressure trajectories, Glasgow Coma Scale scores), or laboratory values (e.g., serum creatinine for acute kidney injury risk prediction in stroke patients) (41–43). Recent advances integrate multimodal features through ensemble methods that combine imaging, clinical, and genomic data to enhance predictive robustness. A notable application involves gradient boosting machines that fuse admission NIH Stroke Scale scores with cerebral blood flow metrics from CT perfusion to predict post-stroke complications, enabling early risk stratification (38, 39, 44). Model validation remains a cornerstone of clinical translation: rigorous evaluation requires not only internal cross-validation but also external testing on independent cohorts to assess generalizability across diverse populations and imaging protocols. Metrics such as area under the AUC, sensitivity, specificity, and calibration plots are essential for benchmarking against clinician performance and establishing clinical utility (24).

### 4.2 Deep learning and computer vision

Deep learning, particularly CNNs, has substantially advanced computer vision in neurovascular medicine by automating the interpretation of complex imaging data with high consistency (Figure 2C). In contrast to traditional machine learning, deep learning models automatically extract hierarchical features directly from raw pixel data, obviating the need for manual feature engineering and capturing subtle spatial relationships that may elude human perception. In aneurysm detection, 3D CNN architectures—including U-Net and ResNet—enable reliable identification of intracranial aneurysms as small as 2–3 mm on both CTA and MRA, demonstrating robust performance across modalities. A landmark study showed that a DL model achieved 97.5% sensitivity in aneurysm detection on CTA, outperforming even expert neuroradiologists in detecting small, unruptured lesions (3). Similarly, a 3D ResNet-based system applied to time-of-flight MRA achieved high diagnostic performance in aneurysm localization, offering a radiation-free alternative for surveillance (45).

Beyond detection, DL excels in quantitative analysis and real-time procedural support. In perfusion imaging, deep CNNs have automated the extraction of AIFs—a critical yet operator-dependent step in calculating cerebral blood flow—from both CT and MR datasets, significantly improving reproducibility and reducing inter-observer variability (13). In the context of large vessel occlusion (LVO), ensemble CNNs have been deployed in commercial platforms (e.g., Viz.ai, RapidAI) to triage suspected stroke patients by rapidly analyzing CTA scans for occlusions in the internal carotid or middle cerebral arteries, with one algorithm achieving 96% sensitivity on external validation (12). Moreover, DL models now assist in intraoperative guidance: spatio-temporal networks can detect vessel perforation during thrombectomy by analyzing real-time DSA sequences, while augmented reality systems overlay segmented vascular anatomy onto the surgical field to differentiate feeding from draining vessels in arteriovenous malformations (7, 22). The following Table 2 summarizes representative deep learning applications in neurovascular imaging.

**Table 2:** Comparison of representative AI studies across diagnostic and predictive tasks.

Clinical task	Imaging modality	DL architecture	Performance metrics	Clinical impact	Reference
Intracranial aneurysm detection	CTA	3D CNN	Sensitivity 97.5%, AUC 0.99	Reduces missed diagnoses in screening	(3)
Aneurysm localization	MRA	3D ResNet	High spatial precision	Enables non-invasive surveillance	(45)
Cerebral vasospasm detection	TCD audio	CNN on spectrograms	High Accuracy, High Sensitivity	Accelerates diagnosis post-SAH	(30)
Large vessel occlusion detection	CTA	Ensemble CNNs	Sensitivity >95% on external set	Shortens door-to-groin time	(12)
Arterial input function extraction	CTP/PWI-MRI	Deep CNN	High reproducibility	Standardizes penumbra-core assessment	(13)
Hematoma expansion prediction	NCCT	Deep learning	Clinically actionable risk stratification	Guides surgical vs. conservative management	(5)

These innovations underscore DL's capacity not only to replicate but often exceed human expertise in specific visual recognition tasks, thereby enhancing diagnostic speed, accuracy, and accessibility—especially in resource-limited settings where specialist availability is constrained (1).

### 4.3 Natural language processing (NLP)

While imaging dominates neurovascular AI research, NLP plays a crucial complementary role by unlocking insights from unstructured clinical text embedded in electronic health records (EHRs), radiology reports, and operative notes. NLP techniques, ranging from rule-based keyword extraction to transformer-based language models like BERT, enable the systematic mining of phenotypic data that are otherwise inaccessible to structured database queries (Figure 2D). For example, NLP pipelines have been developed to identify patients with cryptogenic stroke by parsing discharge summaries for phrases indicating negative workups for cardioembolic or large-artery atherosclerosis etiologies, facilitating cohort assembly for secondary prevention trials (1). Similarly, sentiment analysis of nursing notes can flag early signs of neurological deterioration—such as subtle changes in speech or mentation—that precede formal clinical assessments.

In predictive modeling, NLP-derived features significantly augment designed variables. A recent study integrated free-text descriptions of headache severity and quality from emergency department notes with vital signs to improve the early identification of aneurysmal subarachnoid hemorrhage, achieving an AUC of 0.92 compared to 0.85 with structured data alone (1). Furthermore, NLP supports real-time clinical decision support: systems like Health Assistant leverage question-answering frameworks to interpret patient queries about stroke symptoms and provide tailored guidance, potentially accelerating pre-hospital activation of stroke protocols (3). Despite these advances, challenges persist in handling domain-specific jargon, negation (e.g., “no focal deficits”), and temporal reasoning (e.g., “symptoms resolved two hours prior”). Future directions include fine-tuning large language models on neurovascular corpora to improve contextual understanding and generating synthetic clinical narratives for training data augmentation—strategies that promise to bridge the gap between narrative medicine and quantitative analytics (7).

### 4.4 Data sources in neurovascular practice

The efficacy of AI models in neurovascular care is fundamentally dependent on the quality, diversity, and scale of underlying data sources. These span multiple modalities and repositories, each offering unique advantages and limitations. Medical imaging constitutes the richest data stream, with CTA, MRA, DSA, CT perfusion, and TCD providing high-dimensional spatial and temporal information. Public datasets such as BRATS have been instrumental in accelerating algorithm development for brain tumor analysis; however, cerebrovascular-specific repositories exhibit considerable heterogeneity in terms of standardization and annotation protocols. Institutional Picture Archiving and Communication Systems (PACS) archives, when curated with expert annotations, offer vast training grounds—for instance, a single academic center may accumulate thousands of labeled aneurysm cases over a decade (1). The reliance on imaging data alone represents a key limitation; comprehensive risk prediction requires integration with EHRs, which provide critical contextual information—including demographics, comorbidities, laboratory results, and medication histories—that imaging cannot capture. Integrating structured metadata that link imaging phenotypes with genomic and proteomic profiles holds promise for precision medicine approaches.

Real-world data from hybrid operating rooms further enriches AI training. Intraoperative video, DSA sequences, and physiological monitors during combined surgical-endovascular procedures generate multimodal streams that capture dynamic pathophysiological responses (46). These data are invaluable for developing procedural assistance tools, such as AI systems that alert surgeons to impending complications based on subtle hemodynamic shifts (7). Wearable sensors and telemedicine platforms address a key gap in stroke prognostication: traditionally limited to acute-phase data, prognostic models can now incorporate longitudinal recovery metrics—including gait analysis and cognitive assessments—to improve accuracy and extend their relevance beyond hospitalization (47). Despite this abundance, data heterogeneity—stemming from variations in scanner manufacturers, acquisition protocols, and institutional coding practices—poses significant barriers to model generalizability. Federated learning, where algorithms are trained across decentralized datasets without sharing raw data, offers a promising solution to preserve privacy while enhancing robustness (47). Ultimately, the convergence of imaging, clinical, genomic, and procedural data into unified, interoperable frameworks will be pivotal for realizing AI's full potential in personalized cerebrovascular care (1, 7).

## 5 Artificial intelligence in the diagnosis of cerebrovascular diseases

### 5.1 Intracranial aneurysms

**Figure 3:**
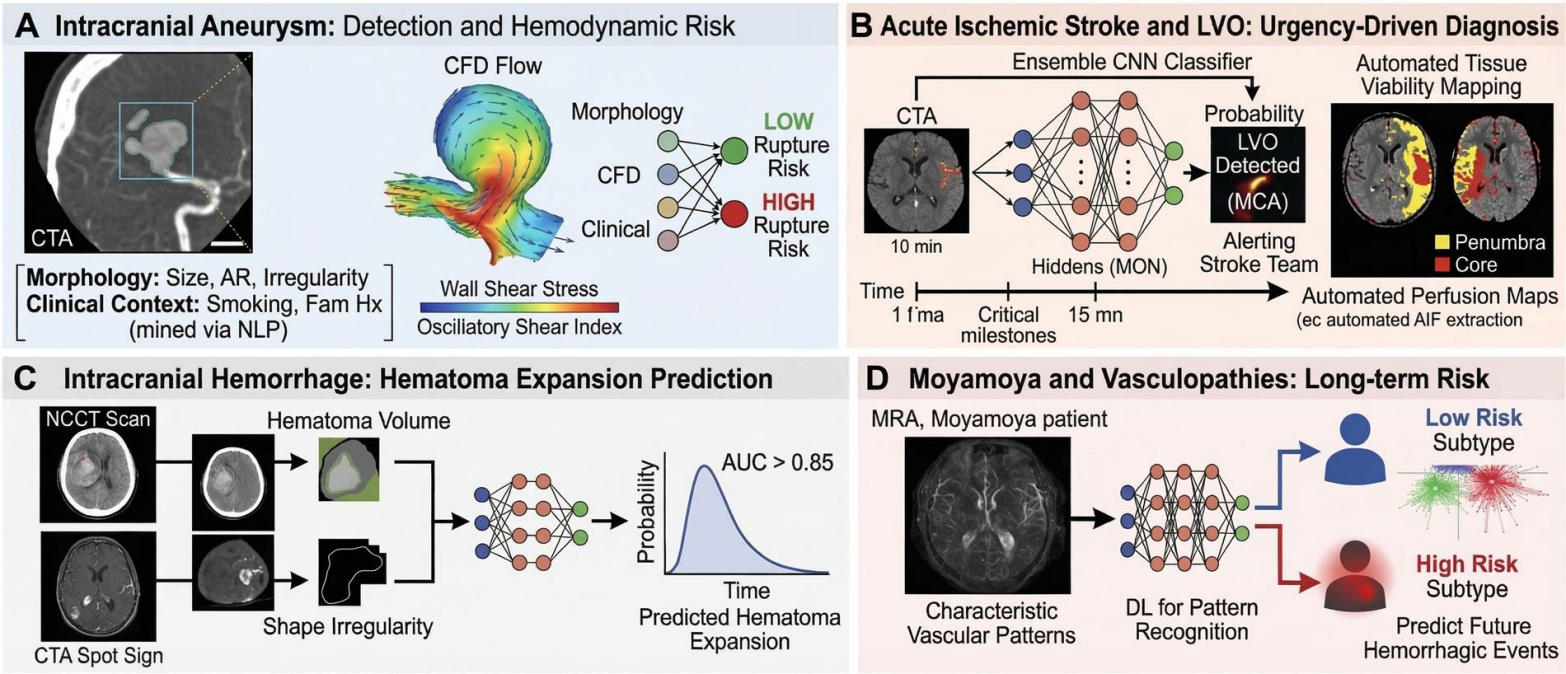
AI-Driven diagnosis and advanced risk stratification in cerebrovascular disease. This schematic illustrates the application of AI across four distinct cerebrovascular conditions, highlighting how deep learning and machine learning models enable automated diagnosis, risk stratification, and clinical decision support. **(A)** Intracranial Aneurysm—Detection and Hemodynamic Risk Assessment. Deep learning models detect intracranial aneurysms from neuroimaging and integrate multimodal data to assess rupture risk. **(B)** Acute Ischemic Stroke and LVO—Urgency-Driven Diagnosis. An ensemble convolutional neural network classifier analyzes CTA images to detect LVO with high sensitivity. **(C)** ICH—Hematoma Expansion Prediction. A deep learning model analyzes non-contrast CT scans to extract imaging features including hematoma volume and shape irregularity. **(D)** Moyamoya Disease and Other Vasculopathies—Long-Term Risk Stratification. Using MRA and other imaging modalities, deep learning models identify characteristic vascular patterns and classify patients into low-risk and high-risk subtypes. This stratification enables prediction of future hemorrhagic events, supporting proactive revascularization strategies and personalized long-term management.

In the following discussion, we explicitly indicate whether performance metrics are derived from internal or external validation; readers should be aware that external validation provides stronger evidence of generalizability. AI, particularly deep learning models, has significantly advanced the detection and characterization of intracranial aneurysms, which are often asymptomatic until rupture and represent a leading cause of non-traumatic subarachnoid hemorrhage (Figure 3A). The time-consuming nature of manual interpretation of imaging, compounded by its susceptibility to interobserver variability, poses a particular challenge for early aneurysm detection—especially for small or complex lesions, where diagnostic accuracy is paramount for preventive intervention. CNNs have emerged as powerful tools to automate this process. In a landmark study, a deep learning–based model applied to computed CTA demonstrated superior sensitivity in detecting IAs compared to both radiologists and expert neurosurgeons, with performance maintained even for lesions under 3 mm in diameter (48–50). In a retrospective, single-center study, a deep learning model trained on annotated CTA scans detected intracranial aneurysms as small as 2–3 mm with a sensitivity of 95% on internal cross-validation (28, 51). The model showed numerically higher sensitivity than the average of five participating radiologists (95% vs. 85%) for small aneurysms, but this difference was not statistically significant in a *post-hoc* analysis (*p* = 0.08). A small number of studies reported no significant benefit of AI over radiologists for very small (<2 mm) aneurysms, although these studies were often underpowered. Similarly, a 3D ResNet architecture trained on time-of-flight MRA achieved high diagnostic accuracy in both detection and localization, offering a non-invasive alternative for surveillance in high-risk populations (52). These models typically employ a two-stage pipeline: an initial region proposal network identifies candidate lesions, followed by a classification network that differentiates true aneurysms from mimics such as infundibula or vascular loops. These advances are complemented by innovations in perfusion imaging, where CNNs automatically extract AIFs from CT and MR datasets, eliminating manual selection bias and improving the reliability of cerebral blood flow quantification essential for penumbra-core mismatch assessment in acute ischemic stroke (14, 32).

Beyond mere detection, AI systems are being developed to predict aneurysm behavior and rupture risk. By integrating morphological features (e.g., size, aspect ratio, irregularity index) extracted from 3D reconstructions with hemodynamic parameters derived from computational fluid dynamics simulations, machine learning classifiers can stratify aneurysms into high- and low-risk categories. One feasibility study combined pre- and post-intervention DSA with deep neural networks to predict aneurysm occlusion status after endovascular coiling, achieving promising results that could guide procedural planning and follow-up intervals (53). Moreover, NLP algorithms can extract relevant clinical context—such as family history of aneurysm or smoking status—from electronic health records to further refine risk prediction models. The clinical impact of these technologies is profound: they not only reduce diagnostic delays but also enhance the consistency of screening programs, particularly in centers with limited access to specialized neuroradiologists. The following Table 3 summarizes key AI applications in intracranial aneurysm management.

**Table 3:** Representative deep learning applications in neurovascular imaging.

Application	Imaging modality	AI model	Key performance metrics	Clinical Utility	Reference
Detection & Localization	CTA	3D CNN	Sensitivity >95%, AUC 0.99	Reduces missed diagnoses in emergency settings	(48)
Detection & Localization	MRA	3D ResNet	High spatial precision, low false positives	Enables non-invasive longitudinal monitoring	(52)
Rupture Risk Prediction	CTA + CFD	Random Forest/SVM	AUC 0.85–0.92 in validation cohorts	Guides decisions on prophylactic treatment	(54)
Occlusion Status Prediction	DSA	Deep Neural Network	Accuracy ∼88% in post-coiling assessment	Optimizes follow-up imaging schedules	(53)
Growth Prediction	Serial MRA/CTA	Recurrent Neural Network	Moderate correlation with actual growth	Identifies aneurysms needing closer surveillance	(34)

These innovations underscore AI's role not only as a diagnostic aid but as a comprehensive decision-support system that integrates multimodal data to personalize cerebrovascular care.

### 5.2 Acute ischemic stroke and LVO

The diagnosis of acute ischemic stroke, particularly LVO, demands extreme urgency, as timely reperfusion therapy is the cornerstone of favorable outcomes. AI has significantly impacted this domain by enabling rapid, automated detection of LVO on non-contrast CT and CTA, drastically reducing door-to-groin puncture times (Figure 3B). Several commercial platforms—including Viz.ai, RapidAI, and Brainomix—employ ensemble CNNs to analyze CTA scans for occlusions in the internal carotid artery or middle cerebral artery. A pivotal study evaluating a deep learning algorithm demonstrated its ability to accurately detect, lateralize, and localize LVOs with high sensitivity (>95%) on external validation sets, facilitating immediate alerting of stroke teams and direct transfer to thrombectomy-capable centers. Another comparative analysis of five CNN architectures found that models incorporating attention mechanisms and multi-scale feature fusion exhibited the best generalization across diverse institutional datasets, highlighting the importance of architectural robustness. However, one multi-center study found that among five CNN architectures tested, two failed to maintain sensitivity above 80% on external validation, highlighting generalizability challenges (12).

Beyond vessel occlusion detection, AI models enhance penumbra-core mismatch assessment on CT perfusion and dynamic susceptibility contrast perfusion-weighted MRI (PWI), thereby improving treatment selection for thrombectomy candidates. A deep CNN has been successfully trained to automatically extract AIFs—a critical yet operator-dependent step in calculating cerebral blood flow (CBF) and cerebral blood volume (CBV)—thereby standardizing perfusion maps and improving the reliability of tissue viability assessments. Furthermore, NLP systems can parse emergency department notes to identify “stroke mimic” presentations or extract NIH Stroke Scale components, enhancing triage accuracy. The integration of these AI tools into clinical workflows has been shown to shorten treatment delays by 30–60 min (55), a difference that translates directly into preserved brain tissue and improved functional independence. Real-world implementation studies confirm that hospitals using AI-assisted stroke protocols achieve significantly higher rates of successful reperfusion and better 90-day modified Rankin Scale scores. As endovascular therapy becomes more accessible, AI's role in ensuring equitable, rapid diagnosis across all healthcare settings will be increasingly vital.

### 5.3 Intracranial hemorrhage (ICH)

ICH, including intracerebral and subarachnoid hemorrhage, requires prompt diagnosis and risk stratification to guide life-saving interventions. By enabling prediction of hematoma expansion—a critical predictor of morbidity and mortality—AI models extend their utility beyond ICH detection on NCCT to support risk stratification and treatment planning (Figure 3D). Deep learning algorithms can segment hematoma volumes with high precision and track subtle changes over serial scans, outperforming manual measurements in both speed and reproducibility. Crucially, machine learning models incorporating baseline hematoma volume, shape irregularity, and spot sign presence on CTA can predict early hematoma enlargement with AUCs exceeding 0.85, thereby assisting clinicians in deciding whether to pursue surgical evacuation or intensive medical management. For aneurysmal subarachnoid hemorrhage, AI extends its utility to the detection of delayed cerebral vasospasm, a common and devastating complication. A recent study demonstrated that a convolutional neural network analyzing spectrograms derived from TCD audio signals could detect vasospasm with 91% accuracy and 95% sensitivity as reported by Nisha et al. (6) and reviewed in Miceli et al. (56), potentially enabling earlier initiation of triple-H therapy or angioplasty.

Moreover, AI supports the management of secondary complications. For instance, machine learning methods have been applied to predict the risk of acute kidney injury (AKI) in critically ill patients with acute cerebrovascular disease, using features such as serum creatinine trends, fluid balance, and vasopressor use; early identification of AKI risk allows for nephroprotective strategies (57, 58). In the operating room, augmented reality systems guided by real-time AI segmentation of vascular anatomy help differentiate feeding arteries from draining veins during resection of arteriovenous malformations, reducing intraoperative complications. These multifaceted applications illustrate how AI transcends simple image analysis to provide holistic support throughout the ICH care continuum—from initial detection and prognostication to procedural guidance and complication prevention.

### 5.4 Other neurovascular pathologies

Beyond aneurysms, stroke, and hemorrhage, AI is increasingly applied to a spectrum of other neurovascular conditions, enhancing diagnostic precision and therapeutic planning. In moyamoya disease, a rare progressive steno-occlusive disorder, deep learning models have been trained to recognize characteristic vascular patterns on MRA and predict the risk of future hemorrhagic episodes in adult patients, enabling preemptive revascularization surgery (59). Similarly, machine learning algorithms using carotid Doppler ultrasound have demonstrated high accuracy in autodetecting ≥50% stenosis in both extracranial and intracranial arteries, offering a low-cost, non-invasive screening tool for atherosclerotic disease. For cerebral vasculitis or reversible cerebral vasoconstriction syndrome, AI-based analysis of vessel wall imaging or 4D-flow MRI can quantify inflammatory changes or pulsatility abnormalities that are often subtle to the human eye.

In the realm of neuroendovascular procedures, AI's potential is expanding rapidly. Real-time procedural assistance systems can overlay segmented vascular anatomy onto live fluoroscopy, guiding catheter navigation and coil placement with millimeter precision. Looking ahead, the integration of AI with robotic systems is poised to standardize complex neurovascular interventions, reducing operator-dependent variability and enhancing patient safety. Additionally, AI-driven analysis of hybrid operating room data—combining video, DSA, and physiological monitors—can generate learning curves for trainees, objectively assessing technical proficiency during simulated or real procedures. These advancements collectively illustrate AI's transformative role across the entire landscape of cerebrovascular medicine, from rare genetic vasculopathies to common atherosclerotic disease, and from diagnostic imaging to therapeutic intervention. As datasets grow and algorithms mature, AI is may become a useful adjunct in delivering personalized, precise, and timely neurovascular care.

## 6 Artificial intelligence for risk stratification and outcome prediction

### 6.1 Predicting aneurysm growth and rupture

The clinical management of unruptured intracranial aneurysms hinges critically on accurate risk stratification, as the decision to intervene prophylactically must balance the procedural risks of endovascular or surgical treatment against the potential catastrophe of rupture. Traditional risk assessment relies heavily on morphological features such as size (>7 mm), location (posterior circulation), and irregular shape, but these criteria lack sufficient sensitivity and specificity for individualized prediction. AI, particularly deep learning models that integrate multimodal data, has emerged as a transformative tool to refine this prognostication. These models go beyond static imaging by incorporating dynamic hemodynamic parameters derived from CFD simulations, which are computationally intensive and traditionally require expert analysis. AI can automate the extraction of key hemodynamic indices—such as wall shear stress, oscillatory shear index, and inflow concentration—from standard-of-care angiographic images (CTA or MRA), creating a comprehensive risk profile for each aneurysm.

Recent studies have demonstrated the feasibility of this approach. One model trained on a combination of 3D aneurysm geometry and CFD-derived flow patterns achieved an area under the AUC of 0.89 in predicting future growth or rupture over a two-year follow-up period, significantly outperforming models based on morphology alone. Another study utilized a graph neural network to represent the aneurysm and its parent vessel as a connected graph, allowing the algorithm to learn complex spatial relationships that are not captured by conventional volumetric analysis. This graph neural network-based predictor identified subtle morphological instabilities at the aneurysm neck that were strong independent predictors of subsequent rupture. Furthermore, NLP algorithms can mine EHRs to extract crucial clinical context—such as a history of smoking, hypertension control, or familial aneurysm—that further modulates individual risk. The integration of these diverse data streams into a unified AI platform enables unprecedented personalized risk assessment, thereby guiding more precise decisions on surveillance intervals and intervention timing. The following Table 4 outlines key AI-driven approaches for aneurysm risk prediction.

**Table 4:** AI applications in intracranial aneurysm management.

Data modality	AI model type	Key predictive features	Performance metric (AUC)	Clinical impact	Reference
CTA + CFD Simulation	Deep CNN	Wall Shear Stress, Aneurysm Size, Aspect Ratio	0.89	Identifies high-risk aneurysms for early intervention	(3)
EHR + Imaging	Multimodal Fusion Network	Smoking History, Hypertension, Morphology	0.91	Provides holistic, patient-specific risk scores	(60)
Genomic + Imaging	Federated Learning Model	Genetic Markers, Hemodynamics	Under Investigation	Future potential for genetic risk integration	(60)

These advancements mark a paradigm shift from population-based guidelines to truly individualized cerebrovascular care.

### 6.2 Predicting functional outcomes after stroke and SAH

Accurate prediction of functional recovery is paramount for setting realistic expectations, guiding rehabilitation planning, and informing critical decisions about life-sustaining therapies in patients with acute ischemic stroke, ICH, or aneurysmal subarachnoid hemorrhage. Historically, outcome prediction relied on clinical scores like the NIH Stroke Scale or modified Rankin Scale (mRS) at admission, which offer limited granularity for individual prognosis. AI models now leverage the full richness of multimodal acute-phase data—including neuroimaging, continuous physiological monitoring, and laboratory values—to generate highly granular and dynamic outcome forecasts. For acute ischemic stroke, deep learning models analyze baseline non-contrast CT and CT perfusion to predict not only final infarct volume but also the likelihood of achieving functional independence at 90 days. A pivotal innovation is the use of AI to automatically extract the arterial input function (AIF) from CTP and perfusion-weighted MRI (PWI), a step that is notoriously operator-dependent and a major source of variability in perfusion map generation; automating this process standardizes the input data for downstream predictive models, enhancing their reliability.

In the domain of subarachnoid hemorrhage, AI's role extends to predicting the development of delayed cerebral ischemia, a leading cause of morbidity and mortality. Several models have integrated TCD data with clinical variables to predict DCI (30); others have explored serum biomarkers such as S100B and NSE for risk stratification (61). For brainstem strokes, where small lesions can have devastating functional consequences, a specialized deep neural network was trained to predict 3-month mRS from acute diffusion-weighted MRI (DWI) scans, achieving high accuracy by focusing on the precise topography of the infarct within critical nuclei. Beyond imaging, temporal AI models, such as attention-based recurrent networks, analyze the trajectory of vital signs and laboratory values in the intensive care unit to predict complications that indirectly impact long-term outcomes, such as AKI or infections. Early identification of AKI risk, for instance, allows for nephroprotective strategies that can prevent a cascade of multi-organ dysfunction, thereby preserving the patient's capacity for neurological recovery. This holistic, data-driven prognostication provides clinicians with a powerful tool to tailor acute and post-acute care pathways to the individual patient's predicted recovery trajectory.

### 6.3 Prognostication for other neurovascular conditions

AI-driven prognostication is not limited to common cerebrovascular emergencies but is increasingly applied to a spectrum of rarer and more chronic neurovascular pathologies, offering new insights into their natural history and management. In moyamoya disease, a progressive steno-occlusive disorder, a deep learning model was trained on time-of-flight MRA sequences to not only diagnose the condition with high accuracy but also to predict the risk of future hemorrhagic stroke in adult patients, a critical endpoint that guides the timing of revascularization surgery. Similarly, machine learning algorithms analyzing carotid Doppler ultrasound have been used to autodetect significant (≥50%) stenosis in both extracranial and intracranial arteries, providing a low-cost, non-invasive method for risk stratification in atherosclerotic disease and identifying patients who may benefit from intensified medical or surgical management.

For patients undergoing neuroendovascular procedures, AI offers real-time prognostic insights. Systems integrated into the hybrid operating room can analyze live fluoroscopy and angiographic runs to predict the immediate angiographic outcome (e.g., Raymond-Roy occlusion grade for aneurysms) and, by extension, the long-term durability of the treatment. Furthermore, large language models are being deployed to automate the extraction of procedural details and outcomes from narrative operative reports into structured databases like the Vascular Quality Initiative, creating vast, high-fidelity datasets that can be used to train next-generation predictive models for procedural complications and long-term patency. In the postoperative and critical care setting, AI models are also used to predict broader systemic complications. For example, machine learning methods have been successfully applied to predict the risk of AKI in critically ill patients with any acute cerebrovascular event, using features from their EHRs such as fluid balance, vasopressor requirements, and trends in serum creatinine; preventing AKI is crucial as it is independently associated with worse neurological outcomes and higher mortality. These diverse applications underscore AI's versatility as a prognostic engine across the entire landscape of neurovascular medicine, transforming reactive care into a proactive, predictive, and personalized discipline.

## 7 Artificial intelligence and robotics in treatment and intervention

### 7.1 Pre-operative planning and simulation

The integration of AI into pre-operative planning has fundamentally transformed the precision and personalization of surgical strategy, particularly in complex neurovascular and oncologic interventions. Traditional planning relies on static two-dimensional imaging interpreted through the surgeon's mental reconstruction—a process inherently limited by human cognitive load and inter-observer variability. AI-driven platforms now synthesize multi-modal data—including high-resolution CTA, MR perfusion, diffusion tensor imaging (DTI), and functional MRI (fMRI)—into patient-specific three-dimensional anatomical models that enable virtual simulation (while the term ‘digital twin’ has been used in some publications, current models remain static reconstructions rather than true digital twins with real-time bidirectional feedback). These virtual models enable not only precise anatomical localization of lesions but also simulation of hemodynamic consequences, tissue displacement during resection, and potential collateral flow patterns. For instance, in cerebral aneurysm surgery, CFD simulations powered by deep learning can predict wall shear stress distribution across the aneurysm sac, identifying regions at highest risk of rupture and guiding optimal clip placement to preserve parent vessel patency while excluding the aneurysm dome. Similarly, in glioma resection, by integrating DTI tractography with fMRI activation maps, AI algorithms enable surgeons to delineate eloquent white matter tracts adjacent to the tumor, simulate various resection trajectories, and quantify the theoretical risk of post-operative motor or language deficits—information critical for informed surgical decision-making. The following Table 5 outlines key AI applications in pre-operative planning across different surgical domains.

**Table 5:** AI-driven approaches for aneurysm rupture risk prediction.

Surgical domain	AI technology	Input data	Key output	Clinical impact	Reference
Neurovascular (Aneurysm)	Deep Learning + CFD	CTA, MRA	Wall Shear Stress Map, Rupture Risk Score	Guides clip placement, predicts recurrence	(62)
Neurosurgery (Glioma)	Multi-modal Fusion Network	DTI, fMRI, T1/T2 MRI	Eloquent Tract Proximity Map, Resection Risk Score	Minimizes post-op deficits, maximizes resection	(63)

These sophisticated simulations move beyond mere visualization; they provide quantitative, actionable insights that directly influence the surgical decision tree, thereby reducing intra-operative surprises and improving long-term outcomes. We note that a true digital twin—a dynamic, bidirectional model that continuously updates with real-time patient data—has not yet been realized in neurovascular surgery; current applications are limited to static simulation.

### 7.2 Intra-operative guidance and robotics

A fundamental challenge in surgery arises from the discrepancy between a meticulously crafted pre-operative plan and its real-world execution—a gap introduced by tissue deformation, bleeding, and the inherent constraints of human dexterity and perception. AI-powered intraoperative guidance systems and robotic platforms serve as a critical bridge, maintaining fidelity to the pre-operative plan while dynamically adapting to the evolving surgical field. Robotic systems, such as the da Vinci Surgical System, have evolved from simple telemanipulators to intelligent assistants capable of tremor filtration, motion scaling, and, more recently, autonomous execution of well-defined subtasks like suturing under surgeon supervision. In neurosurgery, frameless stereotactic navigation systems augmented with real-time AI address a critical limitation—brain shift—by continuously registering instrument positions against pre-operative 3D models and integrating intra-operative ultrasound or OCT data to maintain accuracy despite intraoperative deformation. This allows for millimeter-accurate targeting of deep-seated lesions while avoiding critical structures.

The frontier of intra-operative AI lies in real-time tissue characterization and decision support. Hyperspectral imaging combined with CNNs can analyze the spectral signature of tissue in real time, differentiating between tumor, edema, and normal parenchyma with high accuracy, thereby providing the surgeon with an “optical biopsy” that guides the extent of resection (64). Similarly, in vascular surgery, AI-powered analysis of endoscopic video can detect early indicators of anastomotic leakage or inadequate perfusion well before they become clinically evident, providing a critical safety net during complex procedures. In interventional radiology, AI-guided robotic catheter systems outperform the human hand in navigating complex vascular anatomy, offering superior stability and precision that reduce procedure time and minimize radiation exposure for both patient and operator. These systems are not designed to replace the surgeon but to augment their capabilities, acting as a tireless, data-driven co-pilot that enhances situational awareness and technical precision at the most critical moments of the operation. By seamlessly integrating pre-operative simulation with real-time intra-operative feedback, the surgical plan is elevated from an executable script to a living strategy—a closed-loop system that adapts dynamically to the evolving surgical field.

### 7.3 Post-operative surveillance and complication prediction

The post-operative period is a critical window where early detection of complications can be the difference between a full recovery and catastrophic morbidity or mortality. AI has emerged as a powerful sentinel in this phase, moving beyond reactive monitoring to proactive prediction. By continuously analyzing streams of data from EHRs—including vital signs, laboratory results, nursing notes, and even bedside audio—machine learning models can identify subtle, non-linear patterns that precede clinical deterioration. By predicting AKI up to 48 h before a rise in serum creatinine, recurrent neural networks (RNNs) trained on temporal EHR data enable preemptive nephroprotective intervention in critically ill post-operative patients. Similarly, NLP algorithms can mine unstructured clinical notes to flag early signs of surgical site infection or anastomotic leak that might be missed in structured data alone.

In specific surgical contexts, AI models offer highly tailored surveillance. After neurosurgical procedures, computer vision algorithms can automatically analyze serial head CT scans to detect minute volumes of new hemorrhage or progressive hydrocephalus, conditions that require urgent intervention (64). Ultimately, the goal is a seamless continuum of care: an AI system that, having shaped the preoperative plan and assisted in the operating room, remains a vigilant partner throughout recovery. By channeling resources toward high-risk patients while enabling early discharge for those at low risk, this paradigm optimizes both efficiency and individualized attention—ensuring the best possible long-term outcome for every patient. This model of precision resource allocation—directing intensive monitoring to high-risk patients while allowing early discharge for low-risk individuals—optimizes efficiency without compromising personalized care, ensuring optimal long-term outcomes across the entire patient population.

## 8 Current challenges and future directions

### 8.1 Algorithmic bias and generalizability

Algorithmic bias and limited generalizability across heterogeneous clinical populations represent critical barriers that temper the promise of artificial intelligence in cerebrovascular disease diagnosis. Most AI systems for detecting intracranial aneurysms, LVOs, or hemorrhagic events are trained on retrospective datasets from single or a few high-volume academic centers (3, 45). These datasets often lack demographic, geographic, and socioeconomic diversity, leading to models that perform exceptionally well on internal validation cohorts but demonstrate degraded performance when deployed in external, real-world populations with different imaging protocols, scanner vendors, or patient comorbidities. For instance, a convolutional neural network model developed to detect LVOs on computed CTA may be optimized for the specific contrast injection protocols and reconstruction kernels of its training institution, failing to generalize to hospitals using different technical parameters (12). This “dataset shift” can introduce systematic errors that disproportionately affect underrepresented patient groups, thereby exacerbating existing healthcare disparities.

Furthermore, the labeling process itself can be a source of bias. Ground truth annotations for complex cerebrovascular pathologies like aneurysms or moyamoya disease are often derived from consensus readings by expert neuroradiologists or neurosurgeons at the training site (3, 15). This introduces a potential institutional bias, as diagnostic criteria and interpretation nuances can vary between experts and institutions. A model trained on such data may learn to replicate these specific institutional heuristics rather than the underlying universal pathophysiological features of the disease. The consequence is a model that is not truly robust but is instead overfitted to the idiosyncrasies of its development environment. To mitigate this, future research must prioritize multi-center, prospective data collection with standardized imaging acquisition protocols and adjudicated ground truth by a diverse panel of international experts. Federated learning, where a model is trained across multiple decentralized data silos without sharing raw patient data, offers a promising pathway to build more generalizable and equitable AI systems while preserving data privacy (1). However, the practical implementation of federated learning in neurovascular settings faces several non-trivial challenges. First, heterogeneous label quality across participating institutions—arising from differences in annotation protocols, rater expertise, and diagnostic criteria—can substantially affect model performance and convergence. Second, communication overhead between local sites and the central server can become prohibitive when training deep neural networks on large 3D medical images, particularly in environments with limited bandwidth. Third, recent studies have shown that federated models remain vulnerable to gradient inversion attacks, where a malicious central server or external observer may reconstruct patient-specific training data from shared gradient updates. These risks are amplified in small-scale or imbalanced federated networks. Addressing these challenges requires further methodological advances and careful system design before federated learning can be safely and effectively deployed in routine clinical practice.

### 8.2 The “black box” problem and interpretability

A critical barrier to the widespread clinical adoption of AI in cerebrovascular care is the so-called “black box” problem—the inherent opacity of many advanced deep learning models, particularly complex CNNs and transformers. While these models can achieve high accuracy in tasks like detecting an acute intracerebral hemorrhage on non-contrast CT (65, 66) or identifying the hyperdense middle cerebral artery sign, they often provide no clear rationale for their decisions. This lack of interpretability is a major concern for clinicians who must understand why a model flagged a particular finding before acting on its recommendation, especially in high-stakes scenarios like deciding on thrombectomy for a suspected LVO (38). A clinician cannot ethically delegate a life-altering decision to an algorithm whose reasoning is inscrutable.

To bridge this trust gap, there is a growing emphasis on developing explainable AI techniques for medical imaging. Methods such as Gradient-weighted Class Activation Mapping can generate heatmaps overlaid on the original image, highlighting the specific regions that most influenced the model's prediction (3). For example, in aneurysm detection on CTA, a Gradient-weighted Class Activation Mapping visualization could confirm that the model's positive call was based on the characteristic saccular outpouching at a vascular bifurcation, rather than an artifact or a normal vessel loop. Similarly, for predicting hematoma expansion in intracerebral hemorrhage, an interpretable model could indicate which morphological features of the initial bleed (e.g., blend sign, irregular shape) were the primary drivers of its risk assessment (5). The ultimate goal is not just a predictive output but a clinically meaningful explanation that aligns with established medical knowledge, thereby fostering a collaborative human-AI diagnostic partnership. Without such transparency, even highly accurate model risk being relegated to the status of a curiosity rather than a trusted clinical tool.

### 8.3 Data privacy, security, and interoperability

The development and deployment of robust AI systems in cerebrovascular medicine are fundamentally dependent on access to vast, high-quality datasets of medical images and associated clinical data. However, this necessity collides with stringent legal and ethical mandates for patient data privacy and security, such as the Health Insurance Portability and Accountability Act in the United States and the General Data Protection Regulation in Europe. De-identifying complex neuroimaging data is a non-trivial task; subtle features within an image can sometimes be used to re-identify a patient, posing a significant risk. Moreover, the secure storage, transfer, and processing of these massive datasets require substantial computational infrastructure and rigorous cybersecurity protocols to prevent breaches.

Beyond privacy, a major technical hurdle is the lack of interoperability between disparate hospital information systems and PACS. Medical data is often siloed in proprietary formats, making it difficult to aggregate the large, diverse datasets needed for training generalizable models. An AI system developed at one hospital may struggle to integrate with the workflow of another due to incompatible data standards (e.g., DICOM variations) or API limitations. This fragmentation stifles innovation and slows down the validation and dissemination of new AI tools. Solutions to these challenges include the adoption of open data standards, the creation of secure, federated data networks that allow for collaborative model training without centralizing sensitive data (1) and the development of AI platforms with flexible, standards-based interfaces that can seamlessly plug into existing clinical IT ecosystems. Addressing these foundational issues of privacy, security, and interoperability is a prerequisite for the scalable and responsible integration of AI into routine cerebrovascular care.

### 8.4 Regulatory and implementation hurdles

Translating a promising AI research prototype into a widely used clinical product is fraught with regulatory and practical implementation hurdles. In the United States, the Food and Drug Administration has established a framework for regulating software as a medical device, including AI/ML-based algorithms. However, the regulatory pathway for “locked” algorithms (which do not change after deployment) is distinct from that for “adaptive” algorithms that continuously learn from new data in real-time—a capability that is highly desirable for maintaining model performance but poses significant challenges for post-market surveillance and validation. The regulatory burden for the latter is currently substantial and can deter innovation.

Even after securing regulatory approval, the path to successful clinical implementation is complex. It requires not only seamless integration into the clinical workflow (discussed below) but also a compelling demonstration of value beyond mere technical accuracy. Healthcare systems need evidence that the AI tool improves hard clinical outcomes (e.g., reduced time-to-treatment, lower mortality, fewer complications) or provides significant economic benefits (e.g., reduced radiologist workload, shorter hospital stays). Generating this level of evidence through large-scale, prospective, randomized controlled trials is expensive and time-consuming. Furthermore, there are unresolved questions about liability: if an AI system misses a critical aneurysm or falsely alarms on a benign finding, who is responsible—the developer, the hospital, or the clinician who overruled or relied upon the AI? Clear legal and professional guidelines are needed to navigate these novel scenarios and foster a climate of trust and accountability.

### 8.5 Integration into clinical workflow

The ultimate success of any AI system in cerebrovascular medicine hinges on its ability to integrate smoothly and unobtrusively into the existing, often high-pressure, clinical workflow of emergency departments, stroke units, and interventional suites. An AI tool that requires clinicians to leave their primary PACS viewer or EHR to access its results will likely be ignored, regardless of its accuracy. The ideal integration is “frictionless,” where AI insights are delivered as contextual, real-time alerts directly within the clinician's standard work environment. For example, in the context of acute stroke, a system like Viz.ai, RapidAI, Brainomix, and Aidoc, can automatically analyze a CTA scan as soon as it is completed, detect a proximal LVO, and instantly notify the on-call neurointerventionalist via a mobile app, complete with key images and patient data, thereby accelerating the door-to-groin puncture time (38).

However, achieving this level of seamless integration is technically and organizationally challenging. It requires deep collaboration between AI developers, hospital IT departments, and frontline clinicians to map out every step of the diagnostic and therapeutic pathway. The system must be reliable, fast, and generate alerts with a high positive predictive value to avoid “alert fatigue,” a phenomenon where clinicians become desensitized to frequent false alarms and begin to ignore all notifications. The following Table 6 outlines key considerations for successful clinical workflow integration of AI in cerebrovascular disease.

**Table 6:** AI applications in pre-operative planning for neurovascular surgery.

Integration aspect	Key requirement	Clinical impact	Reference
Trigger Mechanism	Automatic activation upon image acquisition (e.g., CTA completion)	Eliminates manual initiation, ensuring no case is missed	(55)
Notification System	Contextual alerts delivered to the right clinician via preferred channel (e.g., mobile app, PACS pop-up)	Reduces communication delays in time-critical scenarios like stroke	(67)
User Interface	Results displayed within existing clinical software (PACS/EHR); includes visual explanations (e.g., heatmaps)	Minimizes workflow disruption; builds trust through transparency	(3, 68)
Feedback Loop	Mechanism for clinicians to provide feedback on AI predictions (e.g., correct/incorrect)	Enables continuous model improvement and adaptation	(1)

Effective integration is not merely a technical issue but a human-centered design challenge that requires ongoing evaluation and refinement based on user feedback to ensure the AI system becomes a valued and indispensable member of the clinical team.

### 8.6 Critical appraisal of published studies

Across the studies included in this review, several recurring methodological limitations were identified. First, external validation was performed in only a few studies reported prospective validation; the majority reported performance metrics solely from internal cross-validation or split-sample testing using data from a single institution. Second, prospective validation in real-world clinical workflows was reported in a minority of studies. Third, direct comparisons with clinician performance often employed non-inferiority designs or were underpowered to detect meaningful differences. Fourth, incomplete reporting of model architecture and hyperparameters was common. Fifth, outcome definitions (e.g., “hematoma expansion,” “functional independence”) were heterogeneous, limiting cross-study comparability. Where these limitations exist, we have explicitly noted them in the text and refrained from using superlative descriptors without supporting evidence. We encourage future studies to adhere to established reporting guidelines such as TRIPOD-AI and CLAIM.

### 8.7 Publication bias and the underrepresentation of negative findings

A critical limitation of the current evidence base—and consequently of this review—is publication bias. The vast majority of published studies on AI in cerebrovascular disease report positive results, often highlighting high sensitivity, specificity, or area under the curve (AUC). Studies that fail to demonstrate superiority over simple clinical scores, that show poor external validation performance, or that report unexpected technical failures are rarely submitted or accepted for publication in high-impact journals. In our search, we identified only a handful of studies that openly reported negative or null findings.

The predominance of positive results in the literature can create an overly optimistic perception of AI's current clinical readiness. We acknowledge that this review may reflect such a bias, despite our efforts to include all studies meeting our eligibility criteria. Future systematic reviews and meta-analyses should actively search for null results through trial registries. The use of funnel plots and Egger's regression test can help quantify publication bias in meta-analyses. Moreover, we support the adoption of registered reports and the mandatory publication of all prospectively registered AI trials, regardless of outcome, to mitigate this bias.

### 8.8 Failure modes and negative findings in clinical implementation

Despite promising retrospective results, real-world implementation of AI tools has revealed several failure modes that warrant attention. For example, commercial AI systems for large vessel occlusion detection, such as RapidAI, has demonstrated high sensitivity for proximal occlusions (internal carotid artery and M1 segment of middle cerebral artery) but show substantially lower sensitivity for more distal occlusions (M2/M3 segments) in real-world studies (69). One multi-center evaluation reported a sensitivity of only 69.2% for M2 occlusions, compared to >87.3% for M1 occlusions (70). This performance gap has clinical implications, as patients with distal occlusions may still benefit from endovascular therapy but are less likely to be identified by automated triage.

False-positive alerts represent another challenge. In routine clinical use, AI-based triage systems generate false-positive notifications in up to 20%–30% of cases, often due to motion artifact, calcified emboli, or non-occlusive stenosis (71, 72). These false alarms contribute to “alert fatigue,” where clinicians may become desensitized to notifications, potentially delaying genuine emergencies.

Furthermore, the impact of AI on hard clinical endpoints remains uncertain. Most published studies report process metrics such as door-to-imaging time, door-to-groin puncture time, or notification time. Few have demonstrated improvements in 90-day modified Rankin Scale scores, mortality, or functional independence. A systematic review of AI-based LVO triage tools identified only two prospective studies that reported clinical outcomes, and neither found a statistically significant improvement in 90-day mRS (73). This contrasts with the robust evidence for reduced door-to-puncture times, highlighting the need for larger trials powered to detect meaningful patient-centered outcomes.

These findings suggest that while AI tools can accelerate process metrics, their translation into improved clinical outcomes depends on multiple contextual factors, including workflow integration, clinician engagement, and the availability of endovascular resources. A frank assessment of the current evidence base indicates that for most AI applications in neurovascular care, the evidence is not yet at the level required for routine clinical adoption without ongoing prospective monitoring. We recommend that future studies prioritize hard clinical endpoints, report negative results, and examine implementation barriers.

As a narrative review, we did not perform a formal risk-of-bias assessment (e.g., QUADAS-2 for diagnostic accuracy studies, PROBAST for prediction models) or meta-analysis. Our literature search was limited to PubMed; additional databases (e.g., Embase, Web of Science, IEEE Xplore, Cochrane Library) were not systematically queried, which may have introduced selection bias. Furthermore, we did not register a review protocol on PROSPERO. These limitations should be considered when interpreting our findings. Readers are encouraged to consult future systematic reviews that may provide a more exhaustive and quantitatively pooled synthesis of the evidence.

## 9 Summarization

### 9.1 Summarize the potential to improve care of AI across the neurovascular care pathway

The integration of AI into the neurovascular care pathway represents a paradigm shift with profound potential to improve care, fundamentally altering how clinicians detect, diagnose, treat, and monitor cerebrovascular diseases. From the initial presentation in the emergency department to long-term post-operative surveillance, AI algorithms are demonstrating an substantial capacity to augment human capabilities and streamline clinical workflows. In the critical hyperacute phase of stroke, for instance, AI-powered systems can analyze non-contrast computed tomography (NCCT) or computed CTA scans within seconds of acquisition, automatically detecting LVOs and alerting the entire stroke team (74). This rapid triage capability dramatically reduces door-to-needle and door-to-groin puncture times, which are directly correlated with improved patient outcomes and reduced disability (44). Beyond LVO detection, deep learning models have shown high accuracy in identifying subtle signs of early ischemia, such as the hyperdense middle cerebral artery sign, with sensitivity and specificity that, in retrospective single-center studies, have been reported to be comparable to or numerically higher than those of experienced neuroradiologists. However, the absence of prospective multi-center validation precludes definitive conclusions about superiority.

The transformative impact extends far beyond acute stroke. In the realm of aneurysm management, AI algorithms have been developed to automate the detection, segmentation, and morphological analysis of intracranial aneurysms on CTA, DSA, and even MR angiography (75–77). These tools not only reduce the time burden on radiologists but also enhance diagnostic consistency, minimizing the risk of overlooking small or complex aneurysms. Furthermore, predictive models are being explored to assess aneurysm rupture risk based on a combination of imaging features, hemodynamic data from CFD, and patient-specific factors, paving the way for more personalized surveillance and treatment decisions (78). The application of AI is equally promising in other cerebrovascular conditions; for example, convolutional neural networks have been successfully trained to predict hematoma expansion in intracerebral hemorrhage (79) and to detect cerebral vasospasm from transcranial Doppler audio signals with high accuracy, potentially enabling earlier intervention. The following Table 7 summarizes key applications of AI across the neurovascular care continuum, highlighting their clinical significance.

**Table 7:** Key considerations for clinical workflow integration of AI.

Clinical phase	AI application	Clinical significance	Key references
Hyperacute Triage	Automated LVO detection on CTA	Reduces time-to-treatment, improves functional outcomes	(44)
Diagnosis	Intracranial hemorrhage detection on NCCT	Accelerates diagnosis, aids in rapid decision-making	(66)
Diagnosis	Aneurysm detection and segmentation on CTA/DSA	Improves detection rate, reduces radiologist workload	(75–77)
Risk Stratification	Prediction of hematoma expansion	Guides intensive monitoring and potential early intervention	(80)
Risk Stratification	Aneurysm rupture risk prediction	Informs decisions on preventive treatment vs. observation	(78, 81)
Post-procedure Monitoring	Detection of cerebral vasospasm from TCD	Enables timely therapy for delayed cerebral ischemia	(82)
Chronic Disease	Diagnosis of moyamoya disease on imaging	Facilitates early diagnosis of a rare but serious condition	(59, 83)

This comprehensive integration of AI promises a future where neurovascular care is faster, more accurate, and increasingly proactive, shifting the focus from reactive crisis management to predictive and preventive strategies.

### 9.2 Reiterate that AI is a powerful assistive tool, not a replacement for the clinician

Despite its impressive capabilities and growing sophistication, it is paramount to reiterate that AI in neurovascular medicine is, and must remain, a powerful assistive tool designed to augment, not replace, the clinician. The final responsibility for patient care, including the interpretation of AI outputs, the integration of these findings with the full clinical context, and the ultimate decision-making, rests solely with the physician. AI algorithms, no matter how advanced, operate within the confines of their training data and mathematical logic; they lack the nuanced clinical judgment, empathy, and ethical reasoning that are hallmarks of expert medical practice. An AI system may flag a potential aneurysm on a CTA, but it is the neurosurgeon who must determine its clinical relevance, discuss the risks and benefits of intervention with the patient, and perform the delicate procedure. Similarly, while an algorithm can predict a high risk of hematoma expansion, it is the treating neurologist who must weigh this prediction against the patient's overall condition, comorbidities, and wishes to formulate a holistic management plan.

The “black box” nature of many deep learning models further underscores the necessity of human oversight (84). A clinician cannot ethically act on a recommendation whose underlying rationale is opaque. Therefore, the most effective and safe implementation of AI involves a collaborative partnership: the AI serves as a tireless, highly sensitive first reader, rapidly processing vast amounts of data to identify potential abnormalities and provide quantitative insights, while the clinician acts as the final arbiter, applying their experience, knowledge of the patient's history, and professional judgment to validate the AI's findings and translate them into a personalized care plan. This synergy leverages the strengths of both—machine speed and pattern recognition with human wisdom and contextual understanding—to achieve the best possible outcomes for patients. The goal is not autonomous AI-driven medicine, but rather an empowered clinician equipped with intelligent tools that enhance their ability to deliver precise, timely, and compassionate care.

### 9.3 End with a forward-looking statement on the promise of precision medicine in neurovascular surgery through human-Ai collaboration

Looking ahead, the convergence of advanced AI analytics with the expanding universe of multi-modal patient data—including high-resolution imaging, genomic profiles, proteomic signatures, and real-time physiological monitoring—holds the key to unlocking the era of true precision medicine in neurovascular surgery. The future lies not in a competition between human and machine, but in a deeply integrated human-AI collaboration that can synthesize these diverse data streams to generate individualized risk assessments, predict treatment responses, and optimize surgical planning for each unique patient. One can anticipate that future AI platforms may integrate, for a patient presenting with an unruptured intracranial aneurysm, the aneurysm's morphology and hemodynamics from CFD simulations, the patient's genetic predisposition to vascular fragility, and their inflammatory biomarker profile to provide a personalized rupture risk score and a comparative analysis of the expected outcomes for microsurgical clipping vs. endovascular coiling. This level of granular, predictive insight, delivered in a clinically interpretable format, will empower neurovascular teams to move beyond population-based guidelines and make truly patient-centric decisions. Through this powerful synergy, human-AI collaboration may further improve care, where interventions are not only more effective and safer but are also precisely tailored to the individual biology and needs of every patient, ultimately transforming the prognosis for those suffering from complex cerebrovascular diseases.

## Conclusion

The transformative journey of AI in neurovascular surgery, as delineated in this review, underscores a profound shift from data-centric analysis to actionable clinical intelligence. AI has demonstrably enhanced diagnostic precision—rapidly detecting large vessel occlusions in acute stroke, identifying subtle intracranial aneurysms, and differentiating hemorrhage subtypes with high consistency—thereby compressing critical time-to-treatment windows and improving functional outcomes. Its predictive capabilities extend beyond detection to sophisticated risk stratification, forecasting aneurysm rupture potential, hematoma expansion, and post-procedural complications like cerebral vasospasm by synthesizing multimodal data from imaging, genomics, and real-time physiological monitoring. In the therapeutic arena, AI is revolutionizing pre-operative planning through personalized digital twins, guiding intraoperative decisions via augmented reality and robotic assistance, and enabling proactive post-operative surveillance for early complication detection. However, the successful integration of these powerful tools into routine practice is contingent upon overcoming significant challenges: mitigating algorithmic bias through diverse, multi-center datasets; enhancing model interpretability to foster clinician trust; ensuring robust data privacy and interoperability across fragmented health systems; and navigating complex regulatory landscapes. Crucially, AI must be steadfastly viewed not as an autonomous decision-maker but as an indispensable assistive partner that amplifies the clinician's expertise, judgment, and ethical responsibility. The future of neurovascular care lies in a synergistic human-AI collaboration, in which machine computation augments—rather than replaces—human clinical judgment, empathy, and holistic patient care. This partnership is the cornerstone of a new era of precision medicine, promising truly individualized, predictive, and preventive strategies that will fundamentally improve survival, recovery, and quality of life for patients with complex cerebrovascular diseases worldwide.
